# Tabulæ Anatomicæ Osteologiæ

**Published:** 1885-12

**Authors:** 


**Tabulæ Anatomicæ Osteologiæ.** Editæ a Carolo H. von Klein, A. M., M. D. Editio Emendata. Cincinnati, O.: Cincinnati Lithographic Co. 1885.

This is a beautiful and "useful anatomical hand atlas in Latin." The author offers "no apology" for presenting this entirely in Latin, nor should he. Every physician should understand Latin sufficiently to be able to read with ease that contained in any atlas. This is a very complete, as well as beautiful, atlas of osteology. It contains thirty-two tables of plates on a scale of one-half to one-tenth the natural size. The plates are finely executed. The printing is plain and in good-sized type, and is all that could be desired. The paper and binding are fair. What more could we say in praise of the work? It certainly should find its way into the hands of many students and physicians. The author states that it will be sold for a trifle. This will enable all to own a complete atlas, which formerly was not the case.

